# Mutation Scanning Using MUT-MAP, a High-Throughput, Microfluidic Chip-Based, Multi-Analyte Panel

**DOI:** 10.1371/journal.pone.0051153

**Published:** 2012-12-17

**Authors:** Rajesh Patel, Alison Tsan, Rachel Tam, Rupal Desai, Nancy Schoenbrunner, Thomas W. Myers, Keith Bauer, Edward Smith, Rajiv Raja

**Affiliations:** 1 Oncology Biomarker Development, Genentech Inc., South San Francisco, California, United States of America; 2 Chemistry and Innovation Technology, Pleasanton, California, United States of America; 3 Program in Core Research Roche Molecular Systems Inc., Pleasanton, California, United States of America; Dartmouth, United States of America

## Abstract

Targeted anticancer therapies rely on the identification of patient subgroups most likely to respond to treatment. Predictive biomarkers play a key role in patient selection, while diagnostic and prognostic biomarkers expand our understanding of tumor biology, suggest treatment combinations, and facilitate discovery of novel drug targets. We have developed a high-throughput microfluidics method for mutation detection (MUT-MAP, mutation multi-analyte panel) based on TaqMan or allele-specific PCR (AS-PCR) assays. We analyzed a set of 71 mutations across six genes of therapeutic interest. The six-gene mutation panel was designed to detect the most common mutations in the *EGFR*, *KRAS*, *PIK3CA*, *NRAS*, *BRAF*, and *AKT1* oncogenes. The DNA was preamplified using custom-designed primer sets before the TaqMan/AS-PCR assays were carried out using the Biomark microfluidics system (Fluidigm; South San Francisco, CA). A cross-reactivity analysis enabled the generation of a robust automated mutation-calling algorithm which was then validated in a series of 51 cell lines and 33 FFPE clinical samples. All detected mutations were confirmed by other means. Sample input titrations confirmed the assay sensitivity with as little as 2 ng gDNA, and demonstrated excellent inter- and intra-chip reproducibility. Parallel analysis of 92 clinical trial samples was carried out using 2–100 ng genomic DNA (gDNA), allowing the simultaneous detection of multiple mutations. DNA prepared from both fresh frozen and formalin-fixed, paraffin-embedded (FFPE) samples were used, and the analysis was routinely completed in 2–3 days: traditional assays require 0.5–1 µg high-quality DNA, and take significantly longer to analyze. This assay can detect a wide range of mutations in therapeutically relevant genes from very small amounts of sample DNA. As such, the mutation assay developed is a valuable tool for high-throughput biomarker discovery and validation in personalized medicine and cancer drug development.

## Introduction

Biomarkers have assumed a central role in oncology, enabling the detection, characterization, and targeted treatment of a range of cancer types [1]. The successful application of targeted anticancer therapies depends on the detection of disease subtypes that are most likely to respond to treatment. As such, the detection and validation of tumor biomarkers is critical for the ongoing development of personalized healthcare, both through the support of effective and robust drug trials, and the effective employment of targeted therapies in the clinic [2].

Biomarkers are classified according to their utility: diagnostic biomarkers are indicators of biological status that allow classification of tumors according to their genetic and/or phenotypic characteristics. Predictive biomarkers allow the response to a particular line of treatment to be anticipated, based on the known mode of action of the chosen therapy and an understanding of the underlying tumor biology. Prognostic biomarkers enable the prediction of disease progression in the absence of treatment, and have been used to identify signaling pathways that are potential drivers of disease, and putative drug targets [3].

Although techniques such as tissue microarray immunohistochemistry (IHC) and reverse-transcription polymerase chain reaction (RT-PCR) allow high-throughput screening of protein and mRNA biomarkers in clinical samples [4], significant challenges remain. Biomarker levels vary across human populations, and significant heterogeneity may be observed within single cancer types, even within samples from a single tumor [5], [6]. This is exacerbated by the possibility that first-line chemotherapy may induce DNA damage in tumor cells, leading to changes in biomarker status; as biopsy samples are often obtained before first-line treatment, this may be an obstacle to the correct selection of subsequent targeted therapies, although the extent of this effect remains unclear [6].

While some anticancer therapeutics are entering the clinic with companion diagnostic tests, a wider characterization of tumor gene expression and mutation status will enable targeted therapies to be combined for specific patient groups without multiple biopsy procedures. A deeper understanding of different tumor subtypes will help explain mechanisms of drug resistance and open up new channels of therapy and research. For this reason, “biomarker pipelines” play an important role in the development of molecular targeted therapies [7].

There are additional challenges associated with biomarker identification using clinical samples containing poor-quality or degraded DNA in limited quantities. Most clinical samples are formalin fixed and paraffin embedded (FFPE) for preservation and storage. While enabling samples to be archived for subsequent biomarker identification and comparison with patient outcomes, this method of preservation leads to nucleic acid fragmentation and cross-linking, so only a small proportion of sample DNA can be probed successfully [8]. Traditional methods of biomarker detection require 0.5–1 µg high-quality DNA and results may take a significant amount of time to analyze, particularly if samples are to be screened for multiple mutations.

We have developed a high-throughput method for mutation detection (MUT-MAP, mutation multi-analyte panel) based on TaqMan and allele-specific PCR (AS-PCR) assays using a microfluidic chip-based technology. This approach allows the rapid analysis of 71 mutations across a panel of six genes of therapeutic interest. Parallel analysis of 92 clinical trial samples can be carried out using miniscule amounts of DNA (2–100 ng, based on the quality of genomic DNA [gDNA] isolated), allowing the simultaneous detection of multiple mutations in a single sample. DNA can be isolated from both fresh frozen and FFPE samples, and the analysis is routinely completed in 2–3 days.

The six-gene panel mutation assay was designed to detect the most common mutations found in *EGFR*, *KRAS*, *PIK3CA*, *NRAS*, *BRAF*, and *AKT1*. Activating mutations in these genes cause aberrant cell signaling and are found in various types of cancer; their encoded proteins are therefore targets for therapeutic inhibition. For example, mutations in *EGFR* are linked with increased activation of the epidermal growth factor receptor (EGFR) signaling pathway, which drives tumor growth and promotes survival in several types of cancer [9]. The *EGFR* and *KRAS* mutation status is predictive of response to anti-EGFR-targeted therapies such as erlotinib, gefitinib [10], and cetuximab [11]. Additionally, the BRAF inhibitor vemurafenib is only effective in patients with *V600* mutation-positive melanoma [12], [13], and the phosphoinositide-3-kinase (PI3K) inhibitor GDC-0941 is most effective in preclinical tumor models with *PIK3CA* mutations [14].

Although next-generation parallel sequencing holds great promise for mutation detection across the whole genome, these technologies are not yet mature enough for routine, high-throughput analysis of precious clinical samples. Parallel sequencing generally requires larger quantities of DNA for analysis and takes longer to generate data in comparison with our approach. The MUT-MAP microfluidics system provides a readily available platform for the exploratory detection of predictive and prognostic biomarkers in support of current and future personalized healthcare.

## Materials and Methods

### Overview of the MUT-MAP Microfluidics System

Mutation screening with the MUT-MAP microfluidics system is a multi-stage process. First, DNA is preamplified using custom-designed primer sets for the exons/genes of interest. The BioMark platform (Fluidigm Corp.) is then used to conduct a combination of quantitative PCR (qPCR) mutation detection assays. We employ two assay formats for mutation detection: both formats utilize TaqMan detection of the amplified product [15]. In one format, which we refer to as TaqMan genotyping or, simply, TaqMan, the discrimination between mutant and wild-type is driven by a differentially-labeled mutant- and wild-type-specific probe [16]. In the other assay format, the discrimination is driven by a mutant-specific primer, or allele-specific PCR (AS-PCR [17], [18]). The AS-PCR assays incorporate the use of an engineered Thermus species Z05 DNA polymerase (AS1) and, in some cases, covalently modified primers to enhance the specificity of allele-specific qPCR [19], [20].

**Figure 1:**
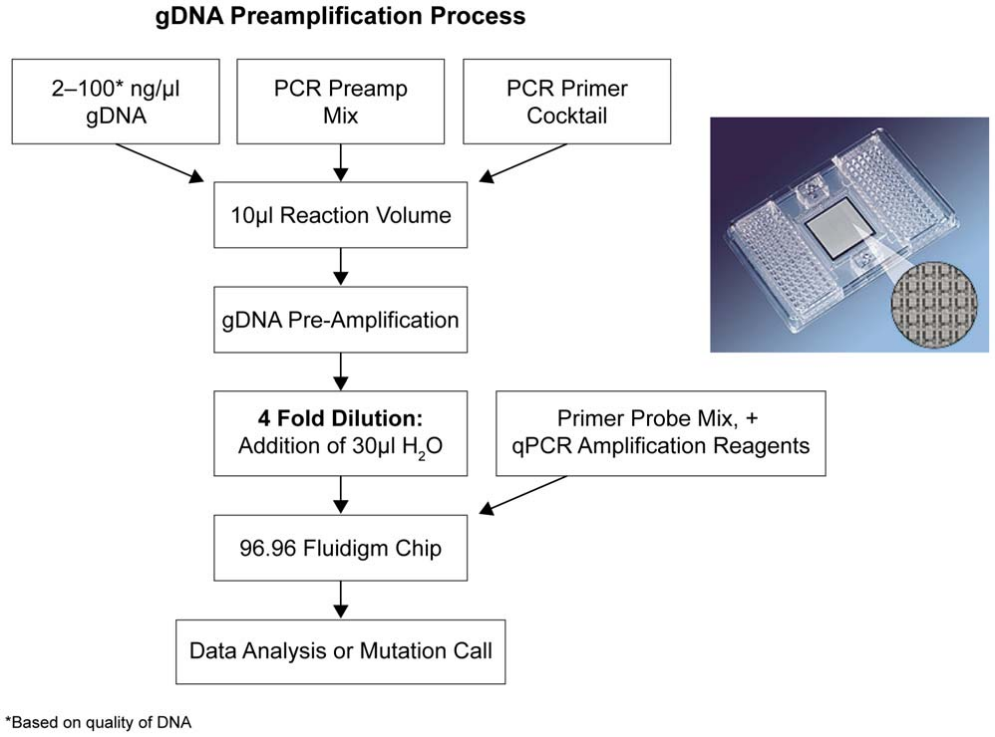
High-Throughput Mutation Detection, Workflow, and Protocol.

The AS-PCR assays were used for *KRAS* and *EGFR* mutation analysis, and have broader coverage of the predominant mutations in these two genes compared with some commercially available assays. An overview of the protocol and process flow is presented in figure 1.

The BioMark protocol involves the introduction of premixed qPCR reagents and preamplified DNA onto the MUT-MAP assay chip via the sample inlets. Assay-specific TaqMan primer/probe mixes are normally added via assay ports. This protocol was modified due to the presence of primers and probes in the qPCR reagents for some reactions (EGFR Mutation Test; Roche Molecular Systems, Inc. [RMS]; Pleasanton, CA). To ensure compatibility with the BioMark platform, these samples were introduced via the assay inlets, and both TaqMan and AS-PCR assay reagents were added via the sample inlets on the microfluidic chip. Data analysis was also modified to accommodate these changes.

### DNA Preamplification

DNA was preamplified in 10 µl reactions on a 96-well plate using a preamplification primer cocktail (Table S1) in the presence of 1x ABI PreAmp Master Mix (Applied Biosystems; Foster City, CA). gDNA (2–10 ng) was isolated from cell lines and fresh frozen samples. However,due to the poor quality of DNA obtained from FFPE clinical samples, 50–100 ng was used for preamplification from this source. Primer concentrations were 100 nM during the amplification reaction. Each preamplification sample set included a gDNA control to determine preamplification performance as well as a no-template control. An additional positive control was made in bulk by preamplification of a cocktail of relevant mutant plasmids for all six genes; this control was run on every chip.

Samples were preamplified using a Tetrad Thermal Cycler (BioRad; Hercules, CA) according to the following protocol: 95°C for 10 minutes, then thermal cycling (20 cycles, each of 15 seconds at 95°C followed by 2 minutes at 60°C). Samples were diluted fourfold, mixed, centrifuged at 3500 rpm (5810 R; Eppendorf; Hauppauge, NY), and stored at 4°C or –20°C until further processing. Following preamplification, rigorous procedures were followed to prevent sample contamination, including the use of dedicated workspaces and pipettes for pre- and post-PCR reaction set-up, laminar flow hoods, and personal protective equipment.

### Preparation of Reagents

Primer/probe concentrations of 900/200 nM were used in the TaqMan reactions to detect mutations in the *PIK3CA*, *BRAF*, *NRAS*, and *AKT* genes. Custom AS-PCR assays (Roche Molecular Systems) were used to detect mutations in *KRAS* and *EGFR* genes along with custom wild-type assays for both genes. A complete description of primers and probes for the TaqMan reactions is presented in table S2.

A commercially available EGFR Mutation Test (Roche Molecular Systems) was modified to achieve compatibility with the two-color BioMark readout (FAM and VIC) for detection of mutations in *EGFR*. Hexachlorofluorescein (HEX)-labeled probes were spiked into kit mastermixes to detect *S768I* and *T790M* in the VIC channel. Additionally, a custom fourth tube was designed to separately detect exon 20 insertion mutations using MMX3 from the RMS EGFR Mutation Test. The *KRAS* allele-specific assays utilized a research kit from Roche Molecular Systems.

Both TaqMan and AS-PCR assays were carried out using the AS1 qPCR master mix. Rox dye (final concentration 55 nM) for signal normalization and 20x gel electrophoresis sample loading buffer (Fluidigm Corp.) were added to the qPCR reactions.

Assays along with AS1 qPCR master mix were run in duplicate by loading 5 µl into each well of the primed 96.96 Fluidigm Chip. The diluted preamplified DNA samples were mixed with equal volumes of 2x DNA assay loading buffer (Fluidigm Corp.). The samples were run by loading 5 µl into each well on the chip. The chip was then placed in the integrated fluidic circuit controller and loaded before analysis with the BioMark reader. The following thermal cycling protocol was used: 50°C (2 minutes), 70°C (30 minutes), 25°C (10 min), 50°C (2 minutes), and 95°C (4 minutes). This was followed by 40 cycles of 95°C (10 seconds) and 61°C (30 seconds). The initial cycle [50°C (2 minutes), 70°C (30 minutes), 25°C (10 minutes)] is part of the protocol recommended by Fluidigm for the 96.96 chip to ensure sufficient mixing of the reagents.

Data were analyzed and cycle threshold (C_T_) values were determined using BioMark real-time PCR analysis software (Fluidigm Corp.), and automated mutation calling was carried out using an algorithm based on the change in C_T_ (ΔC_T_) values between wild-type and mutant or between control and mutant, for TaqMan and AS-PCR assays, respectively.

### Six-Gene Mutation Panel

The use of MUT-MAP in this study allowed the screening of 71 mutations across the *EGFR*, *KRAS*, *PIK3CA*, *NRAS*, *BRAF*, and *AKT1* genes. The mutation coverage of this panel is presented in tables 1 and 2. Validation of mutations detected in clinical samples was performed using commercial mutation detection assays (Qiagen DxS assays for *PIK3CA*, *KRAS*, and *EGFR* mutations), and in-house developed and validated TaqMan assays (for *BRAF*, *NRAS*, and *AKT1*).

**Table 1:** Mutation Coverage Breakdown by Gene.

Six-Gene Mutation Coverage by TaqMan and Prototype *EGFR* and *KRAS* AS-PCR Assays						
Gene	Mutation Count	Exon	Mutation ID	cDNA Mutation Position		Amino Acid Mutation Position
*EGFR*	43	18	6252		2155 G>A	G719S
			6253		2155 G>T	G719C
			6239		2156 G>C	G719A
		19	See table 2 for EGFR exon 19 deletion mutation coverage			
		20	6241		2303 G>T	S768I
			12376		2307_2308 ins 9(gccagcgtg)	V769_D770insASV
			13558		2309_2310 complex(ac>ccagcgtggat)	V769_D770insASV
			12378		2310_2311 ins GGT	D770_N771insG
			13428		2311_2312 ins 9(gcgtggaca)	D770_N771insSVD
			12377		2319_2320 ins CAC	H773_V774insH
			6240		2369 C>T	T790M
		21	6224		2573 T>G	L858R
			12429		2573–2574 TG>GT	L858R
			6213		2582 T>A	L861Q
*PIK3CA*	4	9	760		1624 G>A	E542K
			763		1633 G>A	E545K
		20	775		3140 A>G	H1047L
			776		3140 A>T	H1047R
*KRAS*	18	2	522		35 G>C	G12A
			516		34 G>T	G12C
			521		35 G>A	G12D
			517		34 G>A	G12S
			518		34 G>C	G12R
			520		35 G>T	G12V
			512		34_35 GG>TT	G13D
			532		38 G>A	G12F
			533		38 G>C	G13A
			527		37 G>T	G13C
			529		37 G>C	G13R
			528		37 G>A	G13S
			534		38 G>T	G13V
		3	554		183 A>C	Q61H
			555		183 A>T	Q61H
			549		181 C>A	Q61K
			553		182 A>T	Q61L
			552		182 A>G	Q61R
*BRAF*	1	15	476		1799 T>A	p.V600E
*NRAS*	4	2	564		38 G>A	p.G13D
			580		181 C>A	p.Q61K
		3	584		182 A>G	p.Q61R
			583		182 A>T	p.Q61L
*AKT1*	1	4	33765		49 G>A	p.E17K

## Results

### Plasmid Validation

A series of validation experiments was carried out to confirm the reproducibility and accuracy of the microfluidic assay panel. In order to validate the discrimination of closely related sequences by the mutation screening panel, a complete cross-reactivity analysis was conducted by screening every mutant plasmid target against every mutant-specific assay. The C_T_ values were generated by the BioMark real-time PCR analysis software (Fluidigm Corp.) and plotted as shown in tables 3, 4, and 5. A C_T_ value of 30.0 represents no reactivity, and is indicative of the absence of that allele from the sample. Deviations from this baseline represent assay reactivity, with a lower C_T_ value indicative of increased reactivity. The C_T_ values generated by mutant-specific assays on their corresponding mutant plasmid targets are highlighted in boxed cells (Tables 3, 4, and 5).

**Table 2:** Mutation Coverage for EGFR Exon 19 Deletions.

*EGFR* Exon 19 Deletion Mutations Covered byPrototype *EGFR* AS-PCR Assays			
Mutation Count	Mutation ID	cDNA Mutation Position	Amino Acid Mutation Position
30	26038	2233_2247del15	K745_E749del
	13550	2235_2248>AATTC	E746_A750>IP
	6223	2235_2249del15	E746_A750del
	13552	2235_2251>AATTC	E746_T751>IP
	13551	2235_2252>AAT	E746_T751>I
	12385	2235_2255>AAT	E746_S752>I
	12413	2236_2248>AGAC	E746_A750>RP
	6225	2236_2250del15	E746_A750del
	12728	2236_2253del18	E746_T751del
	12678	2237_2251del15	E746_T751>A
	12386	2237_2252>T	E746_T751>V
	12416	2237_2253>TTGCT	E746_T751>VA
	12367	2237_2254del18	E746_S752>A
	12384	2237_2255>T	E746_S752>V
	18427	2237_2257>TCT	E746_P753>VS
	12422	2238_2248>GC	L747_A750>P
	23571	2238_2252del15	L747_T751del
	12419	2238_2252>GCA	L747_T751>Q
	6220	2238_2255del18	E746_S752>D
	6218	2239_2247del9	L747_E749del
	12382	2239_2248TTAAGAGAAG>C	L747_A750>P
	12383	2239_2251>C	L747_T751>P
	6254	2239_2253del15	L747_T751del
	6255	2239_2256del18	L747_S752del
	12403	2239_2256>CAA	L747_S752>Q
	12387	2239_2258>CA	L747_P753>Q
	6210	2240_2251del12	L747_T751>S
	12369	2240_2254del15	L747_T751del
	12370	2240_2257del18	L747_P753>S
	13556	2253_2276del24	S752_I759del

**Table 3:** Cross-Reactivity of *AKT1*, *BRAF*, *PIK3CA*, and *NRAS* Mutants.

Assays	Plasmid controls *AKT1*, *BRAF*, *PIK3CA*, and *NRAS*										Controls	
	Ak_E17K	Br_V600E	Pk_E542K	Pk_E545K	Pk_H1047R	Pk_H1047L	Nr_Q61K	Nr_Q61R	Nr_Q61L	Nr_G12D	gDNA	NTC
**RNaseP**	30.0	30.0	30.0	30.0	30.0	30.0	30.0	30.0	30.0	30.0	**11.5**	30.0
**AKT_WT**	30.0	30.0	30.0	30.0	30.0	30.0	30.0	30.0	30.0	30.0	**15.8**	30.0
**AKT_E17K**	**20.0**	30.0	30.0	30.0	30.0	30.0	30.0	30.0	30.0	30.0	30.0	30.0
**Br_WT**	30.0	30.0	30.0	30.0	30.0	30.0	30.0	30.0	30.0	30.0	**13.1**	30.0
**Br_V600E**	30.0	**22.4**	30.0	30.0	30.0	30.0	30.0	30.0	30.0	30.0	30.0	30.0
**Pk_E542_WT**	30.0	30.0	30.0	20.6	30.0	30.0	30.0	30.0	30.0	30.0	**15.5**	30.0
**Pk_E542K**	30.0	30.0	**16.6**	30.0	30.0	30.0	30.0	30.0	30.0	30.0	30.0	30.0
**Pk_E545_WT**	30.0	30.0	15.3^a^	30.0	30.0	30.0	30.0	30.0	30.0	30.0	**12.9**	30.0
**Pk_E545K**	30.0	30.0	30.0	**17.2**	30.0	30.0	30.0	30.0	30.0	30.0	30.0	30.0
**Pk_H1047_WT**	30.0	30.0	30.0	30.0	30.0	20.0	30.0	30.0	30.0	30.0	**12.0**	30.0
**Pk_H1047R**	30.0	30.0	30.0	30.0	**15.7**	19.1^a^	30.0	30.0	30.0	30.0	30.0	30.0
**Pk_H1047_WT**	30.0	30.0	30.0	30.0	30.0	26.5	30.0	30.0	30.0	30.0	**12.1**	30.0
**Pk_H1047L**	30.0	30.0	30.0	30.0	30.0	**16.9**	30.0	30.0	30.0	30.0	30.0	30.0
**Nr_Q61_WT**	30.0	30.0	30.0	30.0	30.0	30.0	30.0	30.0	30.0	30.0	**10.5**	30.0
**Nr_Q61K**	30.0	30.0	30.0	30.0	30.0	30.0	**19.1**	30.0	30.0	30.0	30.0	30.0
**Nr_Q61_WT**	30.0	30.0	30.0	30.0	30.0	30.0	30.0	30.0	30.0	30.0	**10.6**	30.0
**Nr_Q61R**	30.0	30.0	30.0	30.0	30.0	30.0	30.0	**22.0**	30.0	30.0	30.0	30.0
**Nr_Q61_WT**	30.0	30.0	30.0	30.0	30.0	30.0	30.0	30.0	30.0	30.0	**10.6**	30.0
**Nr_Q61L**	30.0	30.0	30.0	30.0	30.0	30.0	30.0	30.0	**17.1**	30.0	30.0	30.0
**Nr_G12_WT**	30.0	30.0	30.0	30.0	30.0	30.0	30.0	30.0	30.0	16.2^a^	**10.5**	30.0
**Nr_G12D**	30.0	30.0	30.0	30.0	30.0	30.0	30.0	30.0	30.0	**16.7**	30.0	30.0

a Cross-reactions between the assays are unidirectional and hence do not interfere with accurate mutation calls.

**Table 4:** Cross-Reactivity of *KRAS* Mutants.

Assays	Plasmid Controls *KRAS*																		Controls	
	Kr_ G12S	Kr_ G12C	Kr_ G12R	Kr_ G12D	Kr_ G12V	Kr_ G12A	Kr G12F	Kr_ G13S	Kr_ G13C	Kr_ G13R	Kr_ G13D	Kr_ G13V	Kr_ G13A	Kr_ Q61K	Kr_ Q61L	Kr_ Q61R	Kr_ Q61Hc	Kr_ Q61Ht	gDNA	NTC
**Kr_cntrl**	**12.1**	**12.2**	**12.1**	**12.0**	**12.1**	**12.4**	**13.1**	**12.5**	**12.6**	**12.0**	**13.3**	**11.6**	**12.3**	**12.9**	**12.4**	**12.6**	**13.0**	**12.9**	**10.4**	30.0
**Kr_G12S**	**14.3**	24.8	28.0	28.3	26.6	25.7	30.0	25.8	27.7	26.1	26.4	25.7	25.8	24.2	24.6	24.7	26.7	27.3	23.9	30.0
**Kr_G12C**	24.9	**14.6**	24.2	29.4	27.9	29.1	17.6^a^	26.9	28.9	27.9	30.0	30.0	29.6	26.4	28.7	28.9	28.5	30.0	25.6	30.0
**Kr_G12R**	28.7	24.2	**14.2**	29.8	30.0	28.2	30.0	30.0	28.9	26.0	30.0	28.2	30.0	28.4	30.0	29.4	29.0	28.3	28.5	30.0
**Kr_G12D**	27.6	28.5	23.8	**13.6**	24.8	28.1	30.0	25.2	25.2	24.5	25.3	24.9	25.7	25.0	24.8	25.0	25.9	27.4	22.9	30.0
**Kr_G12V**	18.2	23.4	25.8	13.7^a^	**13.8**	22.7	20.3	24.2	24.3	24.8	23.5	23.9	22.9	22.6	23.3	23.1	23.8	23.4	21.0	30.0
**Kr_G12A**	27.4	26.2	21.8	25.0	22.8	**14.1**	30.0	28.6	30.0	24.8	27.1	28.7	26.9	28.6	28.4	27.6	28.1	29.6	25.3	30.0
**Kr_G12F**	30.0	23.4	30.0	30.0	20.0	29.3	**13.1**	30.0	30.0	30.0	30.0	30.0	30.0	28.3	30.0	29.3	30.0	30.0	29.8	30.0
**Kr_G13S**	15.8^a^	22.9	22.1	24.5	22.1	25.0	26.4	**13.3**	23.8	24.7	24.0	22.7	22.9	23.3	23.0	22.8	24.4	24.2	21.4	30.0
**Kr_G13C**	17.3	24.8	24.7	22.4	13.0^a^	22.0	24.1	23.8	**13.3**	22.4	23.9	23.7	23.7	22.7	22.4	23.0	23.1	23.1	20.5	30.0
**Kr_G13R**	24.9	26.6	29.9	27.7	23.3	28.0	29.8	25.9	22.6	**13.3**	28.0	16.9	26.5	30.0	28.6	30.0	28.5	30.0	26.5	30.0
**Kr_G13D**	29.4	29.0	28.2	19.9	23.7	23.9	30.0	30.0	29.1	29.9	**15.0**	25.4	28.1	22.8	22.8	23.3	23.7	23.6	21.4	30.0
**Kr_G13V**	25.5	20.5	26.0	18.5	25.8	26.8	28.0	29.3	24.8	30.0	24.3	**12.9**	20.2	26.2	25.9	25.0	26.7	26.1	23.9	30.0
**Kr_G13A**	23.6	22.1	23.4	17.0	20.2	20.5	24.7	21.6	13.9^a^	20.0	26.7	21.7	**12.9**	21.5	21.3	21.4	22.0	21.9	19.5	30.0
**Kr_Q61K**	26.7	27.8	27.4	26.6	27.3	26.5	27.9	25.5	27.5	25.0	29.1	25.2	25.8	**15.9**	30.0	30.0	30.0	30.0	25.3	30.0
**Kr_Q61L**	28.2	28.9	27.3	26.3	28.0	28.4	28.3	26.8	27.9	27.1	29.9	26.4	27.4	28.7	**16.5**	28.6	30.0	29.8	25.4	30.0
**Kr_Q61R**	26.4	26.8	26.8	27.0	27.2	27.4	26.4	25.3	27.3	25.1	28.3	24.6	25.3	28.5	23.6	**15.4**	27.8	27.0	24.2	30.0
**Kr_Q61Hc**	30.0	30.0	29.5	29.6	30.0	30.0	29.6	29.7	30.0	29.3	30.0	29.3	30.0	30.0	30.0	26.6	**17.1**	26.6	27.5	30.0
**Kr_Q61Ht**	28.0	27.4	29.2	27.1	27.0	29.5	28.0	26.2	28.4	25.4	29.6	25.6	25.9	26.8	28.4	26.5	28.8	**16.0**	24.8	30.0

a Cross-reactions between the assays are unidirectional and hence do not interfere with accurate mutation calls.

**Table 5:** Cross-Reactivity of *EGFR* Mutants.

Assays	Plasmid Controls *EGFR*									Controls	
		Eg_ex28	Eg_19del	Eg_S768I	Eg_L858R	Eg_T790M	Eg_L861Q	Eg_G719X	Eg_ins	gDNA	NTC
**Eg_ex20_Cntrl**		30.0	30.0	**15.2**	30.0	**17.4**	30.0	30.0	**14.1**	**11.8**	30.0
**Eg_ex28_Cntrl**		**13.0**	30.0	30.0	30.0	30.0	30.0	30.0	30.0	**10.1**	30.0
**Eg_19del**		30.0	**13.6**	30.0	30.0	30.0	30.0	30.0	30.0	24.0	30.0
**Eg_S768I**		30.0	30.0	**15.0**	30.0	30.0	30.0	30.0	28.9	26.4	30.0
**Eg_L858R**		30.0	30.0	30.0	**18.1**	30.0	30.0	30.0	30.0	27.3	30.0
**Eg_T790M**		30.0	30.0	24.8	30.0	**17.1**	30.0	30.0	30.0	23.0	30.0
**Eg_L861Q**		30.0	30.0	30.0	26.1	30.0	**15.7**	30.0	30.0	23.2	30.0
**Eg_G719X**		30.0	30.0	30.0	30.0	30.0	30.0	**18.5**	30.0	26.5	30.0
**Eg_ins**		30.0	30.0	30.0	30.0	29.5	30.0	30.0	**13.3**	23.0	30.0

Eg_ex20_Cntrl assay detects exon 20. Hence *EGFR* exon 20 plasmids carrying *S7681*, *T790M*, and insertion mutations are detected.

The C_T_ values and cross-reactivities obtained from the plasmid data were instrumental in generating an automated mutation-calling algorithm to detect the presence or absence of mutations in clinical samples for each of the six genes in the panel. Samples were re-run on multiple chips to validate both intra- and inter-chip reproducibility.

In general, all samples were correctly identified with high reproducibility and no confounding cross-reactivity. Where cross-reactivity did occur, it was generally an easily discriminated partial reaction. For example, in the TaqMan assays, the cross-reactivity observed between alleles such as *PIK3CA E545* wild-type and *E542K* can be attributed to cross-reactivity of probes with highly similar sequences. In the *KRAS* AS-PCR assays, cross-reactivity is likely due to sequence content at the 3′ end of the primer sequences. The unidirectional nature of these cross-reactions made it easy to build an algorithm to classify mutation status.

### Validation of Cell Line Samples

For cell lines and clinical samples, gene-specific custom algorithms were written, taking into account the control C_T_ and the mutant C_T_ values. Samples showing ΔC_T_ <6 were classified as positive for the specific mutation.

A series of 51 cell lines was screened to detect mutations across the six genes (Table 6). These mutation calls were compared with published characteristics of these cell lines, from the Catalogue of Somatic Mutations in Cancer (COSMIC) [21].

**Table 6:** Correlation Between Mutation Calls in Cell Lines and Those Reported in the Literature.

		Six-Gene Mutation Panel					
Cosmic ID	Samples	*AKT1*	*BRAF*	*PIK3CA*	*NRAS*	*KRAS*	*EGFR*
1286013	MGH-U3	**E17K**	MND	MND	MND	MND	MND
905954	SK-MEL-28	MND	**V600E**	MND	MND	MND	MND
909747	SW1417	MND	**V600E**	MND	MND	MND	MND
905988	MDA-MB-435	MND	**V600E**	MND	MND	MND	MND
906844	DU4475	MND	**V600E**	MND	MND	MND	MND
908125	MEL-JUSO	MND	MND	MND	**Q61L**	MND	MND
910926	BFTC	MND	MND	MND	**Q61L**	MND	MND
724831	H1299	MND	MND	MND	**Q61K**	MND	MND
905955	SKMEL–2	MND	MND	MND	**Q61R**	MND	MND
909771	THP-1	MND	MND	MND	**G12D**	MND	MND
1018466	BT483	MND	MND	**E542K**	MND	MND	MND
905946	MCF-7	MND	MND	**E545K**	MND	MND	MND
908121	MDA-MB-361	MND	MND	**E545K**	MND	MND	MND
906851	EFM19	MND	MND	**H1047L**	MND	MND	MND
905945	T-47D	MND	MND	**H1047R**	MND	MND	MND
905945	T-47D	MND	MND	**H1047R**	MND	MND	MND
910948	MFM-223	MND	MND	**H1047R**	MND	MND	MND
908122	MDA-MB-453	MND	MND	**H1047R**	MND	MND	MND
909778	UACC-893	MND	MND	**H1047R**	MND	MND	MND
1479574	LS180	MND	MND	**H1047R**	MND	**G12D**	MND
905949	A549	MND	MND	MND	MND	**G12S**	MND
905942	NCI-H23	MND	MND	MND	MND	**G12C**	MND
910546	PSN-1	MND	MND	MND	MND	**G12R**	MND
910702	AsPC-1	MND	MND	MND	MND	**G12D**	MND
908122	SW403	MND	MND	MND	MND	**G12V**	MND
753624	CAPAN-1	MND	MND	MND	MND	**G12V**	MND
724873	NCI-H2009	MND	MND	MND	MND	**G12A**	MND
907790	LoVo	MND	MND	MND	MND	**G13D**	MND
905960	MDA-MB-231	MND	MND	MND	MND	**G13D**	MND
907790	LOVO	MND	MND	MND	MND	**G13D**	MND
687800	NCI-H1650	MND	MND	MND	MND	MND	**19del**
1028938	HCC4006	MND	MND	MND	MND	MND	**19del**
1028936	HCC827	MND	MND	MND	MND	MND	**19del**
1336875	pC9	MND	MND	MND	MND	MND	**19del**
924244	NCI-H1975	MND	MND	MND	MND	MND	**L858R/T790M**
909751	SW48	MND	MND	MND	MND	MND	**G719X**
905934	PC-3	MND	MND	MND	MND	MND	MND
910781	AN3 CA	MND	MND	MND	MND	MND	MND
687804	NCI-H1770	MND	MND	MND	MND	MND	MND
905947	786-O	MND	MND	MND	MND	MND	MND
908471	NCI-H1581	MND	MND	MND	MND	MND	MND
908481	NCI-H2196	MND	MND	MND	MND	MND	MND
909907	ZR-75-30	MND	MND	MND	MND	MND	MND
688015	NCI-H2171	MND	MND	MND	MND	MND	MND
905986	SF-268	MND	MND	MND	MND	MND	MND
749712	HCC1395	MND	MND	MND	MND	MND	MND
749714	HCC1937	MND	MND	MND	MND	MND	MND

MND, mutation not detected.

### Validation with FFPE Samples

The assay was further validated using clinical FFPE samples harboring known mutations in the genes of interest. A series of 33 FFPE tumor biopsy samples were analyzed by the six-gene mutation panel. Results were compared with data from traditional micro-well plate qPCR assays: mutations in *EGFR*, *KRAS*, and *PIK3CA* were confirmed using Qiagen DxS assays whereas mutations in *BRAF*, *NRAS*, and *AKT1* were validated with custom in-house-validated TaqMan assays. Execution of the experiments was notably faster with the multiplex assay than with the traditional methods. The MUT-MAP system also required only 20–100 ng DNA compared with 0.5–1 µg DNA for traditional assays (Qiagen DxS assays) covering the same set of mutations.

A good correlation was observed between the experimental results and the traditional mutation detection assays (Table 7). Where samples were available, all outputs were in agreement. The discrepant sample HP-45416 (lung) was not tested for the *EGFR T790M* mutation as the Qiagen DxS assays did not carry the *T790M* assay at the time of the study, and retesting is not possible due to lack of additional sample material.

**Table 7:** Correlation Between Mutation Calls in FFPE Samples and Those Determined by TaqMan/Qiagen DxS Assays.

Samples	Tissues	Six-Gene Mutation Panel							TaqMan/Qiagen DxS					
		*AKT1*	*BRAF*	*PIK3CA*	*NRAS*	*KRAS*	*EGFR*	*AKT1*		*BRAF*	*PIK3CA*	*NRAS*	*KRAS*	*EGFR*
HP-40263	CO	MND	**V600E**	**H1047R**	MND	MND	MND	MND		**V600E**	_a	MND	MND	MND
HP-41677	CO	**E17K**	**V600E**	MND	MND	MND	MND	_a		**V600E**	MND	MND	MND	MND
HP-30630	CO	MND	**V600E**	**E542K**	MND	MND	MND	MND		_a	**E542K**	MND	MND	MND
HP-29630	CO	MND	**V600E**	**E545K**	MND	MND	MND	MND		_a	**E545K**	MND	MND	MND
HP-31183	NOS	MND	MND	**E545K**	MND	**Q61R**	MND	MND		MND	**E545K**	MND	_a	MND
HP-32064	NOS	MND	MND	**H1047R**	MND	**G12D**	MND	MND		MND	**H1047R**	MND	**G12D**	MND
HP-33002	NOS	MND	MND	**E545K**	MND	**G12C**	MND	MND		MND	**E545K**	MND	**G12C**	MND
HP-30760	NOS	MND	MND	**E545K**	MND	**G12S**	MND	MND		MND	**E545K**	MND	**G12S**	MND
HP-30626	CO	MND	MND	**E542K**	MND	**G12V**	MND	MND		MND	**E542K**	MND	**G12V**	MND
HP-40224	CO	MND	MND	MND	**Q61R**	MND	MND	MND		MND	MND	**Q61R**	MND	MND
HP-41675	CO	MND	MND	MND	**Q61K**	MND	MND	MND		MND	MND	**Q61K**	MND	MND
HP-40253	CO	MND	MND	MND	MND	**G12A**	MND	MND		MND	MND	MND	**G12A**	MND
HP-32864	NOS	MND	MND	MND	MND	**G12C**	MND	MND		MND	MND	MND	**G12C**	MND
HP-44508	CO	MND	MND	MND	MND	**G12D**	MND	MND		MND	MND	MND	**G12D**	MND
HP-40092	CO	MND	MND	MND	MND	**G12D**	MND	MND		MND	MND	MND	**G12D**	MND
HP-30770	NOS	MND	MND	MND	MND	**G12R**	MND	MND		MND	MND	MND	**G12R**	MND
HP-41699	CO	MND	MND	MND	MND	**G12C**	MND	MND		MND	MND	MND	**G12C**	MND
HP-32201	NOS	MND	MND	MND	MND	**G12C**	MND	MND		MND	MND	MND	**G12C**	MND
HP-41676	CO	MND	MND	**E545K**	MND	**G12S**	MND	MND		MND	_a	MND	**G12S**	MND
HP-40264	CO	MND	MND	MND	MND	**G12S**	MND	MND		MND	MND	MND	**G12S**	MND
HP-40122	CO	MND	MND	MND	MND	**G12V**	MND	MND		MND	MND	MND	**G12V**	MND
HP-41713	CO	MND	MND	MND	MND	**G12V**	MND	MND		MND	MND	MND	**G12V**	MND
HP-40249	CO	MND	MND	MND	MND	**G13D**	MND	MND		MND	MND	MND	**G13D**	MND
HP-32375	NOS	MND	MND	MND	MND	**G13D**	MND	MND		MND	MND	MND	**G13D**	MND
HP-45416	LU	MND	MND	MND	MND	MND	**L858R/T790M**	MND		MND	MND	MND	MND	**L858R**
HP-45863	NOS	MND	MND	MND	MND	MND	**L858R**	MND		MND	MND	MND	MND	**L858R**
HP-46155	LU	MND	MND	MND	MND	MND	**19del**	MND		MND	MND	MND	MND	**19del**
HP-44217	NOS	MND	MND	MND	MND	MND	**19del**	MND		MND	MND	MND	MND	**19del**
HP-44217	NOS	MND	MND	MND	MND	MND	**19del**	MND		MND	MND	MND	MND	**19del**
HP-46155	LU	MND	MND	MND	MND	MND	**19del**	MND		MND	MND	MND	MND	**19del**
HP-29847	CO	MND	MND	**E545K**	MND	**G12A**	MND	MND		MND	**E545K**	MND	**G12A**	MND
HP-30384	CO	MND	MND	MND	MND	MND	MND	MND		MND	MND	MND	MND	MND

MND, mutation not detected.

CO, Adenocarcinoma of Colon.

LU, Adenocarcinoma of Lung.

NOS, Not otherwise specified.

_a, Insufficient DNA to complete analysis.

### Sample Input Titrations

In order to confirm the reproducibility and consistency of the methodology, sample input titrations were carried out. To define the effective DNA input concentration over which the assay could be considered accurate, and identify the wild-type and mutant C_T_ values for each gene, DNA input was varied for plasmids, cell lines, and FFPE samples, with sample preamplification (Table 8). The C_T_ values for both the mutant and wild-type show the expected response to input concentration over the titration range.

**Table 8:** Sample Input Titrations: Effect on Assay Performance.

Plasmid DNA	Mutation Status	Fg Plasmid	Wild-type C_T_	Mutant C_T_	
Plasmid #1	Pk_E542K	100	30	12.28	
		10	30	15.71	
		1	30	18.55	
Plasmid #2	Pk_E545K	100	30	13.23	
		10	30	16.23	
		1	30	19.98	
Plasmid #3	Pk_H1047R	100	30	11.02	
		10	30	15.33	
		1	30	19.12	
Plasmid #4	Pk_H1047L	100	30	13.63	
		10	30	17.50	
		1	30	21.37	
**FFPE DNA**	**Mutation Status**	**DNA (ng)**	**Wild-type C_T_**	**Mutant C_T_**	**ΔC_T_**
HP-30770	Kr_G12R	160	10.66	15.87	5.21
		40	12.66	17.88	5.23
		10	14.48	19.99	5.51
HP-30630	Pk_E542K	160	14.81	15.21	0.40
		40	16.64	16.68	0.04
		10	18.60	18.93	0.33
**Cell Line DNA**	**Mutation Status**	**DNA (ng)**	**Wild-type C_T_**	**Mutant C_T_**	**ΔC_T_**
MGH-U3	Ak_E17K	120	12.01	11.44	−0.57
		15	15.23	15.17	−0.06

### Platform Reproducibility Validation

**Figure 2:**
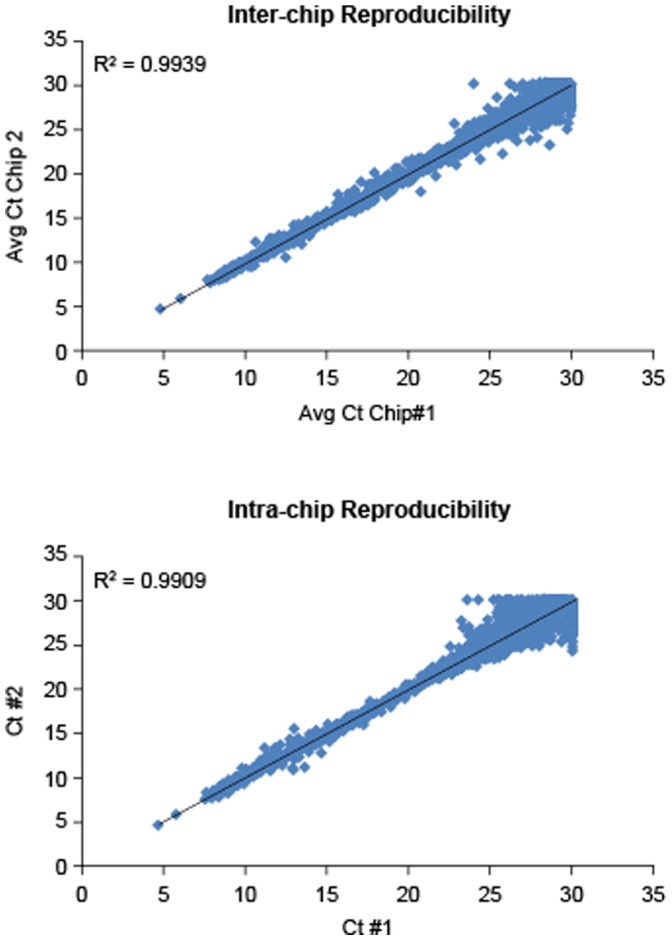
Inter- and Intra-Chip Reproducibility Titrations. The MUT-MAP panel qPCR assays were run in duplicate and C_T_ outputs were plotted to determine both inter- and intra-chip reproducibility. Data for a typical mutation panel run are shown, with R^2^ correlations of 0.9939 and 0.9909 for inter- and intra-chip reproducibility, respectively.

The reproducibility of data from mutation detection assays was also evaluated by the comparison of duplicate experiments. The inter- and intra-chip variability in assay C_T_ values was assessed as shown in figure 2. A total of 5664 duplicate pairs were mapped on a scatter plot, and the Pearson correlation coefficient (*R* ^2^) was calculated. The *R* ^2^ values were found to be over 0.99 for FAM as well as VIC channels, indicating excellent inter- and intra-chip reproducibility of data generated by the assay.

## Discussion

The future of oncology biomarker detection can be delivered by many promising technologies, including multiplexed protein assays, and parallel next-generation genome sequencing [22], [23]. The limited maturity of many of these techniques, combined with their timescale and infrastructure demands, means that there is an unmet need for robust high-throughput biomarker detection methods in the clinical drug development setting.

Our validation has demonstrated that MUT-MAP offers a means of detecting a wide range of mutations in a panel of therapeutically relevant genes, enabling the detection of predictive and prognostic biomarkers from very small amounts of sample DNA. A cross-reactivity analysis showed that this platform has the ability to reliably discriminate between closely related mutations. In addition, the ability of the assay to provide robust reproducible data has been validated in both cancer cell lines and FFPE biopsy samples using considerably smaller amounts of sample DNA than traditional assays. Such an approach enables the study of a wide range of oncogenic mutations in precious clinical samples with very little tissue available for analysis.

As mutations previously thought to be unique to particular tumor types have been shown to be present across a range of cancers (Sanger COSMIC database [24]), the six-gene sample panel used here could be applied to multiple clinical and preclinical studies. The parallel detection of multiple mutations in a single sample also supports biomarker development for combination treatment regimens, where previous analyses would have taken place independently. Parallel analysis also removes the need for sample tracking over multiple assays, which arises with traditional screening methods. The process is further optimized for clinical research and clinical trials by the availability of commercial kit components, facilitating adaptation of this technique to select patients for experimental therapeutic regimens based on gene mutation biomarker combinations which are identified using the multiplex approach.

In addition to biomarker mapping in the clinical setting, MUT-MAP will enable the retrospective analysis of stored FFPE samples, allowing additional data to be obtained from previous studies and possibly identifying previously unknown biomarker associations. The AS-PCR component of the assay uses proprietary primer modifications and an enzyme screened for improved mismatch discrimination. This enables the high level of sensitivity demonstrated in our study and allows us to multiplex allele-specific assays. This sensitivity enables the accurate and reliable identification of mutation status in multiple genes, from poor-quality, low-mass, preserved clinical samples, thereby allowing the maximum amount of data to be obtained from each sample, and repeat experiments to be conducted from the same biopsy. This capability has exciting potential for the future study of low-yield exploratory biomarkers such as circulating tumor DNA [25]. This highly flexible platform can be used to detect mutations beyond the six genes included in this study; in addition, the precise quantification of each amplicon opens up the possibility of being able to detect copy number variations. Most significantly, however, the MUT-MAP assay can form the basis for the development of a platform to support efficient biomarker discovery and validation in support of detection and personalized healthcare.

## Supporting Information

Table S1 **Preamplification Primer Sequences.**
(DOCX)

Table S2 **TaqMan and Mutation Detection Assays.**
(DOCX)
