# Correction: Mutation Scanning Using MUT-MAP, a High-Throughput, Microfluidic Chip-Based, Multi-Analyte Panel

**DOI:** 10.1371/annotation/2bf00719-6566-4ef0-8f75-e4895309b84a

**Published:** 2013-09-09

**Authors:** Rajesh Patel, Alison Tsan, Rachel Tam, Rupal Desai, Jill Spoerke, Nancy Schoenbrunner, Thomas W. Myers, Keith Bauer, Edward Smith, Rajiv Raja

Jill Spoerke should be included in the author byline. She should be listed as the fifth author, and her affiliation is 1: Oncology Biomarker Development, Genentech Inc., South San Francisco, California, United States of America. The contributions of this author are as follows: Conceived and designed the experiments, contributed reagents/materials/analysis tools.

The correct citation is: Patel R, Tsan A, Tam R, Desai R, Spoerke J, et al. (2012) Mutation Scanning Using MUT-MAP, a High-Throughput, Microfluidic Chip-Based, Multi-Analyte Panel. PLoS ONE 7(12): e51153. doi:10.1371/journal.pone.0051153

