# Supplementary material for: Mutation Scanning Using MUT-MAP, a High-Throughput, Microfluidic Chip-Based, Multi-Analyte Panel
Source: PLoS One. 2012 Dec 17;7(12):e51153. doi: 10.1371/journal.pone.0051153 (PMC3524125; doi:10.1371/journal.pone.0051153)
Supplement: Table S1 — Preamplification Primer Sequences. (DOCX) [file pone.0051153.s001.docx]

# Supplementary data

**Table S1.** Preamplification Primer Sequences.

| **Preamplification Primer Sets for Six-Gene Panel** | | |
| --- | --- | --- |
| **Gene** | **Primer** | **Sequence** |
| *PI3K* | P.542.P23 | AGCTAGAGACAATGAATTAAGGGAAA |
|  | P.542.P020R | TCCATTTTAGCACTTACCTGTGAC |
| *PI3K* | P.1047.P28 | TTCGAAAGACCCTAGCCTTAGA |
|  | p.1047.P29 | TGTGTGGAAGATCCAATCCA |
| *KRAS* | KRAS.22F | TTTATTATAAGGCCTGCTGAAAATG |
|  | ESS196 | TTGTTGGATCATATTCGTCCACAA |
| *KRAS* | Kras_61_FC1 | TTCCTACAGGAAGCAAGTAGTAATTGAT |
|  | Kras_61_RC1 | CAAAGAAAGCCCTCCCCAGT |
| *BRAF* | BR_15_FP3 | TATTTCTTCATGAAGACCTCACAGTAA |
|  | BR_15_RP1 | ATCCAGACAACTGTTCAAACTGATG |
| *NRAS* | N.61.P1 | GGTGAAACCTGTTTGTTGGACAT |
|  | N.61.P2 | TGTATTGGTCTCTCATGGCACTGT |
| *NRAS* | N.12.P3 | TTGCTGGTGTGAAATGACTGAGT |
|  | N.12.P4 | CTGGATTGTCAGTGCGCTTTT |
| *EGFR* | Ef_18_FP3-2 | TGGTGAGGGCTGAGGTGAC |
|  | EGFR18_RC1 | CTGTGCCAGGGACCTTACCTTATA |
| *EGFR* | Ef_19_FP3 | ATAGGGACTCTGGATCCCAGAA |
|  | EGFR19_RC1 | ATGAGCAGGGTCTAGAGCAGA |
| *EGFR* | RMSEGFR20_FC1 | GCTTTTCCTCCATGAGTACGTA |
|  | Ef20_R6 | GAGCCAATATTGTCTTTGTGTTCC |
| *EGFR* | Ef_21_FP3 | CTGGCATGAACATGACCCTG |
|  | Ef_21_RP4 | CTGACCTAAAGCCACCTCCTT |
| *EGFR* | EGFR ex28_FP | TGCTTTGCTGATTACTTCACCTCT |
|  | EGFR ex28_RP | GTAGTGTGGGTCTCTGCTGG |
| *AKT1* | P.AKT1.ex4.pre.f | CCTGGCTGCCTGGCGAGGGT |
|  | P.AKT1.ex4.pre.r | GTAGCCAATGAAGGTGCCAT |
| *RNaseP* | ABI RNaseP (FAM) 20x mix |  |
