# Supplementary material for: Mutation Scanning Using MUT-MAP, a High-Throughput, Microfluidic Chip-Based, Multi-Analyte Panel
Source: PLoS One. 2012 Dec 17;7(12):e51153. doi: 10.1371/journal.pone.0051153 (PMC3524125; doi:10.1371/journal.pone.0051153)
Supplement: Table S2 — TaqMan and Mutation Detection Assays. (DOCX) [file pone.0051153.s002.docx]

**Table S2.** TaqMan and Mutation Detection Assays.

| **Gene** | **Base Change** | **Amino Acid** | **Primer/Probe** | **Primer ID** | **Probe Label** | **Sequence** | **Assay** |
| --- | --- | --- | --- | --- | --- | --- | --- |
| *AKT1* | 49G>A | E17K | Forward | AKT1_FP_TAQ |  | GAGGGTCTGACGGGTAGAGTGT | TaqMan |
|  |  |  | Reverse | AKT1_RP_TAQ |  | CCATCATTCTTGAGGAGGAAGTA |  |
|  |  |  | WT | VIC-R-E17 | VIC | GTAGGGGAGTACATCAAG |  |
|  |  |  | Mutant | FAM-R-K17 | 6FAM | GTCTGTAGGGAAGTACATC |  |
| *BRAF* | 1799T>A | V600E | Forward | BRAF.600.F4 |  | TGAAGACCTCACAGTAAAAATAGGTGA | TaqMan |
|  |  |  | Reverse | BRAF.600.R2 |  | ACTGATGGGACCCACTCCATC |  |
|  |  |  | WT | BRAFWT | VIC | TAGCTACAGTGAAATC |  |
|  |  |  | Mutant | BRAFV600E | 6FAM | CTAGCTACAGAGAAAT |  |
|  | Exon 9 | WT | Forward | P.542.23f |  | AGCTAGAGACAATGAATTAAGGGAAA | TaqMan |
|  |  |  | Reverse | P.542.20r |  | CTCCATTTTAGCACTTACCTGTGA |  |
|  |  |  | WT | PI3K_9_Prb1 | 6FAM | TTCTACACGAGATCCTC |  |
| *PIK3CA* | 1624G>A | E542K | Forward | P.542.P1 |  | GCTCAAAGCAATTTCTACACGAGAT |  |
|  |  |  | Reverse | P.542.P2 |  | TGTCTGTGACTCCATAGAAAATCTTTCT |  |
|  |  |  | WT | P.E542.R1 | VIC | CTCTCTCTGAAATCACTGA |  |
|  |  |  | Mutant | P.K542.R2 | 6FAM | CCTCTCTCTAAAATCACTGA |  |
|  | 1633G>A | E545K | Forward | P.545.P4 |  | GCAATTTCTACACGAGATCCTCTCT |  |
|  |  |  | Reverse | P.542.P30 |  | CATTTTAGCACTTACCTGTGACTCCAT |  |
|  |  |  | WT | P.E545.R24 | VIC | TGAAATCACTGAGCAGGAG |  |
|  |  |  | Mutant | P.K545.R25 | 6FAM | TGAAATCACTAAGCAGGA |  |
|  | 3140A>G | H1047R | Forward | P.1047.P7 |  | GGCTTTGGAGTATTTCATGAAACA |  |
|  |  |  | Reverse | P.1047.P8 |  | GAAGATCCAATCCATTTTTGTTGTC |  |
|  |  |  | WT | P.H1047.R8 | VIC | ATGATGCACATCATGGT |  |
|  |  |  | Mutant | P.R1047.R9 | 6FAM | TGATGCACGTCATGGT |  |
|  | 3140A>T | H1047L | Forward | P.1047.P7 |  | GGCTTTGGAGTATTTCATGAAACA |  |
|  |  |  | Reverse | P.1047.P8 |  | GAAGATCCAATCCATTTTTGTTGTC |  |
|  |  |  | WT | P.H1047.R8 | VIC | ATGATGCACATCATGGT |  |
|  |  |  | Mutant | P.L1047.R10 | 6FAM | AATGATGCACTTCATGGT |  |
| *NRAS* | 181C>A | Q61K | Forward | N.61.P1 |  | GGTGAAACCTGTTTGTTGGACAT | TaqMan |
|  |  |  | Reverse | N.61.P2 |  | TGTATTGGTCTCTCATGGCACTGT |  |
|  |  |  | WT | N.Q61.R1 | 6FAM | AGCTGGACAAGAAGA |  |
|  |  |  | Mutant | N.K61.R6 | VIC | CAGCTGGAAAAGAA |  |
|  | 182A>G | Q61R | Forward | N.61.P1 |  | GGTGAAACCTGTTTGTTGGACAT |  |
|  |  |  | Reverse | N.61.P2 |  | TGTATTGGTCTCTCATGGCACTGT |  |
|  |  |  | WT | N.Q61.R3 | 6FAM | CAGCTGGACAAGAA |  |
|  |  |  | Mutant | N.R61.R8 | VIC | TACAGCTGGACGAGAA |  |
|  | 182A>T | Q61L | Forward | N.61.P1 |  | GGTGAAACCTGTTTGTTGGACAT |  |
|  |  |  | Reverse | N.61.P2 |  | TGTATTGGTCTCTCATGGCACTGT |  |
|  |  |  | WT | N.Q61.R3 | 6FAM | CAGCTGGACAAGAA |  |
|  |  |  | Mutant | N.L61.R9 | VIC | ATACAGCTGGACTAGAA |  |
|  | 35G>A | G12D | Forward | N.12.P3 |  | TTGCTGGTGTGAAATGACTGAGT |  |
|  |  |  | Reverse | N.12.P4 |  | CTGGATTGTCAGTGCGCTTTT |  |
|  |  |  | WT | N.G12.R15 | 6FAM | TTGGAGCAGGTGGTGT |  |
|  |  |  | Mutant | N.D12.R19 | VIC | TGGAGCAGATGGTG |  |

WT, wild-type.
